# Investigation of the Role and Effectiveness of Chitosan Coating on Probiotic Microcapsules

**DOI:** 10.3390/polym14091664

**Published:** 2022-04-20

**Authors:** Lóránd Erdélyi, Ferenc Fenyvesi, Bernadett Gál, Ádám Haimhoffer, Gábor Vasvári, István Budai, Judit Remenyik, Ilona Bereczki, Pálma Fehér, Zoltán Ujhelyi, Ildikó Bácskay, Miklós Vecsernyés, Renátó Kovács, Judit Váradi

**Affiliations:** 1 Department of Pharmaceutical Technology, Faculty of Pharmacy, University of Debrecen, Nagyerdei Körút 98, H-4032 Debrecen, Hungary; lorand.erdelyi@gmail.com (L.E.); fenyvesi.ferenc@pharm.unideb.hu (F.F.); g.berni97@gmail.com (B.G.); haimhoffer.adam@pharm.unideb.hu (Á.H.); vasvari.gabor@pharm.unideb.hu (G.V.); feher.palma@pharm.unideb.hu (P.F.); ujhelyi.zoltan@pharm.unideb.hu (Z.U.); bacskay.ildiko@pharm.unideb.hu (I.B.); vecsernyes.miklos@pharm.unideb.hu (M.V.); 2 Doctoral School of Pharmaceutical Sciences, University of Debrecen, H-4032 Debrecen, Hungary; 3 Faculty of Engineering, University of Debrecen, Ótemető Str. 2-4, H-4028 Debrecen, Hungary; budai.istvan@eng.unideb.hu; 4 Institute of Food Technology, Faculty of Agricultural and Food Sciences and Environmental Management, University of Debrecen, H-4032 Debrecen, Hungary; remenyik@agr.unideb.hu; 5 Department of Pharmaceutical Chemistry, University of Debrecen, Egyetem tér 1, H-4032 Debrecen, Hungary; bereczki.ilona@pharm.unideb.hu; 6 Department of Medical Microbiology, Faculty of Medicine, University of Debrecen, H-4032 Debrecen, Hungary; kovacs.renato@med.unideb.hu; 7 Faculty of Pharmacy, University of Debrecen, H-4032 Debrecen, Hungary

**Keywords:** microencapsulation, chitosan, alginate, probiotic, *Lactobacillus*

## Abstract

Microencapsulation and coating are preferred methods to increase the viability of the probiotic strains. The effect of microencapsulation technologies and materials used as microcapsule cores on viability is being investigated during development. In the present study, chitosan-coated and Eudragit L100-55-coated alginate microspheres were produced to encapsulate *Lactobacillus plantarum* probiotic bacteria. After the heat loading and simulated gastrointestinal juice dissolution study, the differences in viability were compared based on the CFU/mL values of the samples. The kinetics of the bacterial release and the ratio of the released live/dead cells of *Lactobacillus plantarum* were examined by flow cytometry. In all cases, we found that the CFU value for the chitosan-coated samples was virtually zero. The ratio of live/dead cells in the 120 min samples was significantly reduced to less than 20% for chitosan, while it was nearly 90% in the uncoated and Eudragit L100-55-coated samples. In the case of chitosan, based on some published MIC values and the amount of chitosan coating determined in the present study, we concluded the reason for our results. It was the first time to determine the amount of the released chitosan coat of the dried microcapsule, which reached the MIC value during the dissolution studies.

## 1. Introduction

Due to concerns about antibiotic use and the appreciation of the role of the microbiome in the gastrointestinal tract in productivity, more and more probiotic products are being developed for animal feed. The use of probiotics may be a suitable alternative to the growth-promoting effect of antibiotics. The main purposes of their use in feed are to improve the growth rate and feed use of the animal and to prevent and control the appearance of enteric pathogens. Increased productivity in livestock can be associated with improved digestion and nutrient absorption [1].

Probiotics are used as a premix in admixture with animal feed. Probiotic cultures are exposed to external effects not only during the preparation of feed but also during the application of compositions, which result in a further reduction in the number of germs [2,3]. When introduced into the digestive tract, probiotic bacteria are exposed to the acidic action of gastric juice and bile acids that reduce the viability of bacterial cells [4,5,6]. Viability can be improved by microencapsulation and with an additional coating [7,8]. The pH tolerance of probiotic-containing microcapsules has been studied in several publications. However, we have less knowledge about the protection of microencapsulation against high temperatures, which is important in the production of feed. Microcapsules are micrometer-sized particles that contain core-embedded probiotics. Efficient, uniform mixing of micromaterial particles with feed is not possible in larger farm animals. In this case, the microcapsules should be added to the feed during extrusion. The extrusion is carried out with hot steam and granules of suitable size are produced under high compression force [9,10,11].

The various *Lactobacillus* strains, *Bifidobacterium bifidum* and *Enterococcus faecium*, are most commonly used as probiotics in feed according to their purpose (e.g., to increase egg production and quality in poultry, and to increase the growth rate in pigs) [12,13]. The selection of probiotics is based on their ability to reach their destination in high germ counts, adhere to mucous membranes and epithelial cells, and survive at low pH values and high bile salt concentrations. The development of microcapsules for animal nutrition purposes is carried out using similar methods and excipients as those used in humans [14,15,16,17]. The destabilizing effect of the same digestive enzymes and gastric juice is also examined in the study of microcapsules; therefore, the results of the research on microcapsules developed for human use can be considered.

The aim of our development is to improve the survival rate of selected probiotic strains by the microencapsulation and coating method, which is significantly reduced during production and application [18,19]. First, we examine the heat tolerance of probiotic strains and compare how microencapsulation and coating alter viability. Only a few results have been published for the study of thermal tolerance; however, survivability increased by microencapsulation in all cases. In the microencapsulation of probiotics, alginate-based microcapsules were mostly prepared [20,21,22,23,24,25], in addition to carrageenan [25], xanthan gum, and gellan gum [19]. Based on the experimental results, the best survival rate and release kinetics were achieved with alginate-based microcapsules, so our experiments were also performed with sodium alginate. Low molecular weight chitosan as a microcapsule coating agent has also been shown to be effective in several published results despite of its antimicrobial activity [25,26].

In addition to chitosan, we also used Eudragit L100-55 as a coating material in our studies. Chitosan is acidic soluble; however, Eudragit L100-55 is dissolved above pH 5.5, as it is an enteric coating agent. The effect of coats with different solubility on the survival rate was investigated in the dissolution studies.

## 2. Materials and Methods

### 2.1. Materials

*Lactobacillus plantarum* subsp. *plantarum* (ATCC 14917) was bought from ATCC (Manassas, VA, USA), and *Bifidobacterium bifidum* and *Enterococcus faecium* was a gift of Judit Remenyik (Institute of Food Technology, University of Debrecen, Debrecen, Hungary). Sodium alginate powder, low viscosity grade, was purchased from Donauchem (Budapest, Hungary). Eudragit L100-55 was purchased from Evonik Industries AG (Niederkassel, Germany). Low molecular weight chitosan (50–190 kDa), phosphate buffered saline (PBS), and FITC were purchased from Sigma-Aldrich (Budapest, Hungary). SYTOX Green Nucleic Acid Stain for flow cytometry were purchased from ThermoFischer Scientific (Budapest, Hungary). Torpac^®^ Gelatin Capsules were ordered from Torpac Europe BV (SG Heerlen, Netherlands). Columbia Blood Agar (CliniChem Ltd., Budapest, Hungary), MRS agar (Merck, Budapest, Hungary), and Bifidobacteria Selective Medium (Merck, Budapest, Hungary) were used for cell viability test.

### 2.2. Preparation and Coating of Alginate Microcapsules

Three types of probiotic microcapsules were prepared separately by using *Lactobacillus plantarum, Bifidobacterium bifidum*, and *Enterococcus faecium*. Probiotic bacterium-loaded alginate microcapsules (ALG) were prepared by the gelation method. Planktonic bacterium suspension (1.5 × 10^9^ CFU/mL) 1 mL was added to 200 g 2 w/w% sterile sodium alginate solution. The vibration nozzle technology was used by Buchi 390-Pro equipment (Buchi, Switzerland). The operating condition was set to 1800 Hz vibration frequency, 1000 V voltage, 150 µm nozzle size, and 4.5–5 mL/min feed flow rate. Microcapsules were allowed to harden in 500 mL 0.1 M calcium chloride solution in 30 min.

The chitosan coating of wet microcapsules was performed by adding 200 mL of 0.4 w/w% chitosan solution. The low molecular weight chitosan was dissolved in 10 V/V% acetic acid solution; every utensil was sterilized. The microcapsules were stirred and incubated for 30 min then washed with 0.1M calcium chloride solution to remove the unbounded chitosan. Wet alginate and chitosan-coated microcapsules were lyophilized to increase their shelf life.

*Lactobacillus plantarum*-loaded microcapsules were coated with Eudragit L100-55 after freeze-drying. Eudragit coating was performed only with *Lactobacillus plantarum*. A Mini-Glatt + micro-kit fluid bed system (Glatt GmbH, Weimar, Germany) was used to coat 10.0 g microcapsule with 20.0 mL 96 V/V% ethanolic Eudragit L100-50 solution (10 w/w%). The bottom-spray process was performed according to the following method. The process variables were kept constant, the inlet air pressure was 0.10 bar, and the product temperature was maintained at 35–37 °C by keeping the inlet airflow temperature at 45 °C. The coating solution was held at ambient temperature and was sprayed through a two-channel nozzle (inner insert diameter 0.3 mm). The peristaltic pump’s flowrate (Flocon 1003; Berlin GmbH; Germany) was 0.81 mL/min and the atomizing air pressure was 0.26 bar. The coated particles were dried until the bed temperature reached 39 °C, while the inlet air temperature was maintained at 45 °C during the coating.

### 2.3. Characterisation of Coated and Uncoated Microcapsules

The morphology of the wet microcapsules was investigated by a light microscope (Zeiss Axio Scope). A1 fluorescent microscope (Carl Zeiss Microimaging GmbH, Göttingen, Germany). The size of microcapsules was analyzed with ZEN 2012 v.1.1.0.0. software (Carl Zeiss Microscopy GmbH, Göttingen, Germany). The morphology of dry microcapsules was characterized by a scanning electron microscope. Before SEM examination, gold-sputtered coating was not deposited on the surface of the samples. The measurement requires a vacuum and a low accelerating voltage of 5 kV. A Bruker EDX 70 detector was used to determine the chemical composition of the samples. Scanning electron microscopy images were taken with a Hitachi Tabletop Microscope 3030 (TM3030) (Hitachi, Tokyo, Japan).

### 2.4. Cell Viability Test

The living cell numbers were determined from previously treated samples using quantitative culturing. Aliquots of 0.1 mL were removed directly after the various treatment; afterwards, samples were serially diluted 10-fold and plated (4 × 0.03 mL) onto Columbia Blood Agar (CliniChem Ltd., Budapest, Hungary), MRS agar (Merck, Budapest, Hungary), and Bifidobacteria Selective Medium (Merck, Budapest, Hungary), for *Enterococcus faecium*, *Lactobacillus plantarum*, and *Bifidobacterium bifidum*, respectively. Agar plates were incubated at 37 °C inside the anaerobe chamber for three days in the case of *Lactobacillus plantarum* and *Bifidobacterium bifidum*, as well as at 37 °C in the presence of 5% CO_2_ for two days in the case of *Enterococcus faecium*. All experiments were performed in triplicate.

### 2.5. Thermotolerance Test under Dry and Wet Heat Treatment

To study the thermal resistance of probiotic bacteria, two methods of dry heat (80, 100 °C) and one method of wet heat (80 °C) were performed. Duration of heat load test at 80 °C dry heat for 10 min, at 100 °C dry heat 5 min, and at 80 °C wet heat 1 min were chosen. The dry heat tests were performed in a dry heat oven. The wet heat tests samples were incubated in a water bath: 30 mg of microcapsules were dispersed in 2 mL of peptone water. The peptone water heat loading test was performed by pipetting 80 °C heated peptone water to the lyophilized formulations, then immersed in a water bath tempered at 80 °C for one minute. After the incubation, samples were cooled in water bath (20 °C), samples were sonicated for 10 s, and CFU/mg were determined. This method was the most suitable to simulate the pelleting conditions.

At the end of the dry heating incubation time, 2 mL of peptone water were added to the microcapsules (30 mg); afterwards, samples were sonicated for 10 s and CFU/mg were determined.

### 2.6. Dissolution Test in Simulated Gastrointestinal Fluids

Three groups of microcapsules (A: uncoated alginate; C: chitosan-coated; E: Eudragit L100-55-coated) were filled into gelatin capsules. Manually, 30 mg of microcapsules were filled into Torpac^®^ Gelatin Capsules (9el size). Gelatin capsules were divided into two further groups, and one of them were used uncoated for the dissolution test. Another group was manually coated with Eudragit. Eudragit L100-55 was dissolved in acetone and 20 w/w% solution was used for coating. The microcapsule-loaded gelatin capsules were immersed into the coating agent and air dried for 4 h.

**Figure 1:**
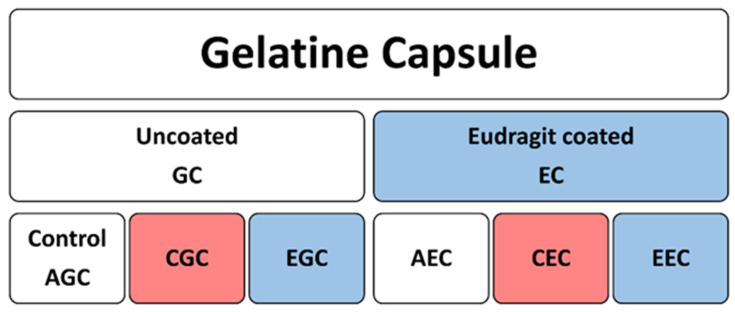
Schematic figure of gelatin capsule samples prepared for the dissolution test. Red color represents chitosan coating, blue represents Eudragit L100-55 coating. GC: uncoated gelatin capsules, EC: Eudragit L100-55 coated gelatin capsules, AGC: uncoated alginate microcapsule filled in uncoated gelatin capsule, CGC: chitosan-coated microcapsule filled in uncoated gelatin capsule, EGC: Eudragit L100-55 coated microcapsule filled in an uncoated gelatin capsule, AEC: alginate microcapsule filled in Eudragit L100-55 coated gelatin capsule, CEC: chitosan-coated alginate microcapsule filled in Eudragit L100-55 coated gelatin capsule, EEC: Eudragit L100-55 -coated alginate microcapsule filled in Eudragit L100-55 coated gelatin capsule.

The dissolution test was performed in simulated gastric (pH 2.0) and intestinal fluids (pH 7.4). The pH of the sterile PBS (pH 7.4) was adjusted to pH 2.0 with phosphoric acid and used as simulated gastric juice. For the experiments, six groups of gelatin capsules were used. AGC was the control group containing uncoated alginate microcapsule filled in an uncoated gelatin capsule (Figure 1). CGC and ECG were chitosan-coated and the Eudragit-coated microcapsule filled in an uncoated gelatin capsule (GC). AEC, CEC, and EEC were the groups of Eudragit-coated gelatin capsules (EC). Each gelatin capsule (at groups *n* = 3) was dissolved in 5 mL of simulated gastrointestinal fluid for 60 min. Capsules were incubated in a shaking incubator at 37 °C, then 500 µL samples were taken, sonicated for 10 s, and CFU/mg was determined. The pH 2.0 medium was removed after 60 min and replaced with simulated intestinal fluid. The intestinal dissolution was performed for 60 min, then 500 µL samples were sonicated for 10 s and CFU/mg were determined.

### 2.7. Cell Release Profiles and Live/Dead Cell Determination from Coated and Uncoated Microcapsules (Number of Cells by Flow Cytometry)

*Lactobacillus* release was investigated in simulated gastric (pH 2.0) and intestinal fluid (pH 7.4). The same amount of uncoated alginate capsules, chitosan-, and Eudragit L-coated microcapsules samples were measured (25 mg), and 500 µL pH 2.0 dissolution medium was added. Samples were incubated in a shaking incubator at 37 °C, and after the 60 min incubation time, the acidic samples (50 µL) were taken. Then, the pH of the acidic dissolution medium was neutralized with 35 µL 2.7 w/w% sodium hydroxide; thus, the pH was set to 7.4. Further samples were taken at 120, 180, and 240 min. After each sample collection, the dissolution medium was replaced with 50 μL. All samples were centrifuged at 1000 rpm for 1 min, the supernatants were collected, and the released bacterial cells were stained with SYTOX Green reagent at 1 µM final concentration for 30 min at 37 °C. Samples were analyzed with Guava Easy Cyte 6HT-2L flow cytometer (Merck Ltd., Darmstadt, Germany). Using green (525/30 nm) and red (695/50 nm) fluorescence channels, cells were gated out on a green versus red dot plot. Released cell numbers and live /dead ratios were evaluated.

### 2.8. Synthesis of FITC-Labeled Chitosan

The FITC-labeled chitosan was synthetized and purified according to a published method [27]. The chitosan solution was prepared by dissolving chitosan in 100 mL of a 0.1 M acetic acid solution at a concentration of 1% w/v. FITC solution (2 mg/mL in methanol, 50 mL) was added to the acidic aqueous solution and then a further 100 mL of dehydrated methanol was mixed. The reaction mixture was kept in the dark for 3 h and the FITC-labelled chitosan was precipitated with 1 L of 1 M NaOH.

The precipitate was filtered and dialyzed with 4 L, and daily renewed with deionized water until FITC was no longer present in the dialysis vessel, which was checked by microplate fluorometer (Fluostar Optima, BMG Labtechnologies Offenburg, Germany) at 492 nm excitation and 520 nm emission wavelengths. The dialysis-purified product was then freeze-dried. The efficiency of the labeling was determined by fluorometer. FITC was dissolved in 50:50 water 6.0 pH/propane-1,2-diol and standard dilutions were made. The maximum fluorescence intensity data were calibrated as a function of FITC concentration, and the molar ratio of free amine to FITC-labeled residues was 7.8:1.

### 2.9. Determination of Dissolved Chitosan Coat

FITC-labeled chitosan was used to quantify the chitosan coating of the microcapsule. The steps for the preparation of the microcapsules and the coating were the same as described in Section 2.2. However, ¼ partially FITC-labelled chitosan was used to prepare the chitosan solution used for coating (200 mL of 0.4 w/w%). The coated microcapsules were dried in a lyophilizer. The dissolution test was performed with the dried microcapsules, the protocol of which is also the same as previously described (Section 2.3). A total of 25 mg of the dried chitosan-coated microcapsule was weighed, and the dissolution was examined in 500 µL of pH 2.0 dissolution medium at 37 °C for 60 min; then, the pH of the dissolution medium was increased to 7.4 and the samples were taken at 240 min. The fluorescence of the samples was examined by fluorometer, and the dilution series of the FITC-labeled chitosan solution was calibrated.

### 2.10. Statistical Analysis

For statistical analyses, GraphPad Prism 5.0 software (GraphPad Software Inc., La Jolla, CA, USA) was used. Data are presented as means ± SD. Comparisons of groups were performed using one-way and two-way ANOVA and Bonferroni multiple comparison test. Differences were considered significant at *p* < 0.05; **** *p* < 0.0001, and ** *p* < 0.01.

## 3. Results

### 3.1. Thermotolerance Test under Dry and Wet Heat Treatment

The improved thermotolerance of the different probiotic bacteria was investigated at three different heat exposure protocols. In all three circumstances, it can be concluded that *Lactobacillus plantarum* and *Bifidobacterium bifidum* showed a similar tendency to their heat load, while *Enterococcus faecium* showed a different susceptibility pattern in the case of uncoated and chitosan-coated microcapsules.

**Figure 2:**
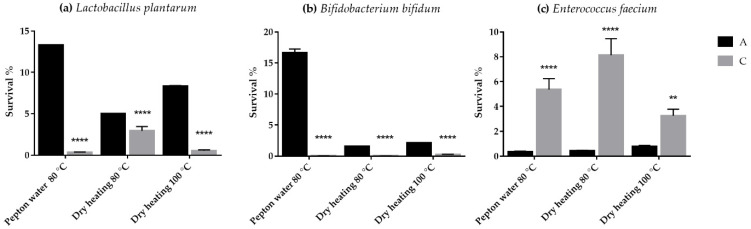
Thermotolerance of *Lactobacillus plantarum* (**a**), *Bifidobacterium bifidum* (**b**), and *Enterococcus faecium* (**c**) loaded in alginate-based uncoated (A) and chitosan-coated (C) microcapsules. Data are presented as means ± SDs, *n* = 3. Differences were considered significant at *p* < 0.05; **** *p* < 0.0001, and ** *p* < 0.01.

The survival rates of *Lactobacillus plantarum* and *Bifidobacterium bifidum* in uncoated microcapsules were 13.33 ± 0.023% and 16.66 ± 0.59%, while 0.33 ± 0.045% and 0.03 ± 0.0085% in chitosan-coated microcapsules after the wet heat load. The survival of *Enterococcus faecium* in uncoated microcapsules were 0.36 ± 0.042%, while 5.36 ± 0.87% in chitosan-coated microcapsules after wet heat load (Figure 2a–c).

### 3.2. Characterization of Coated and Uncoated Microcapsules

**Figure 3:**
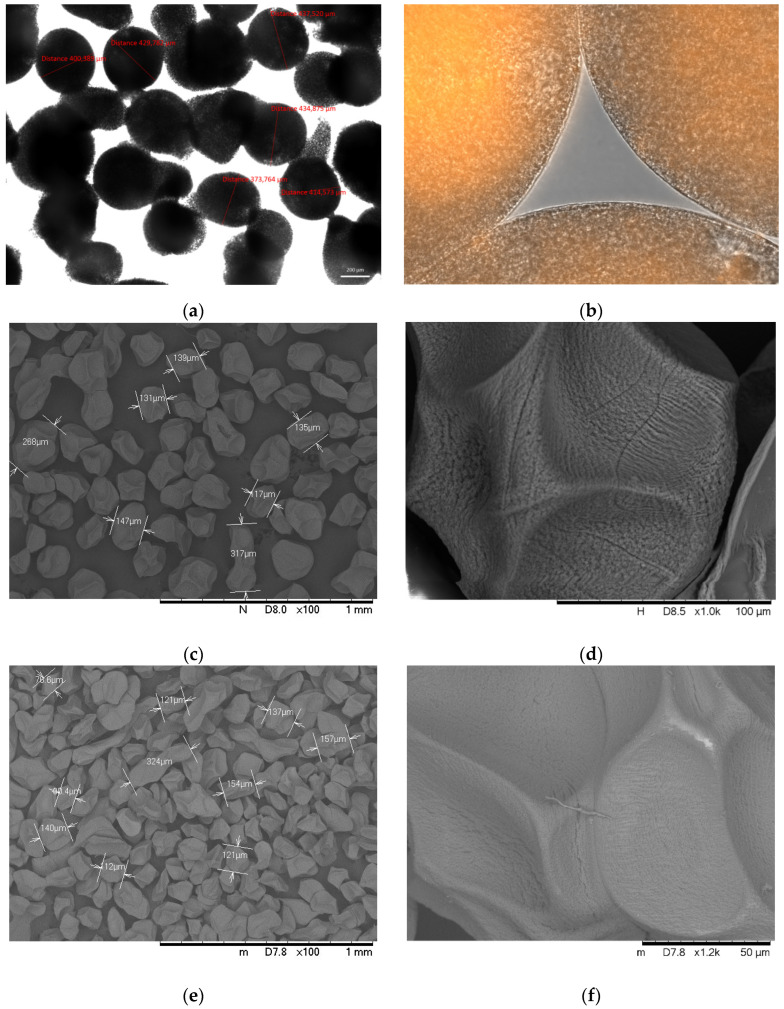

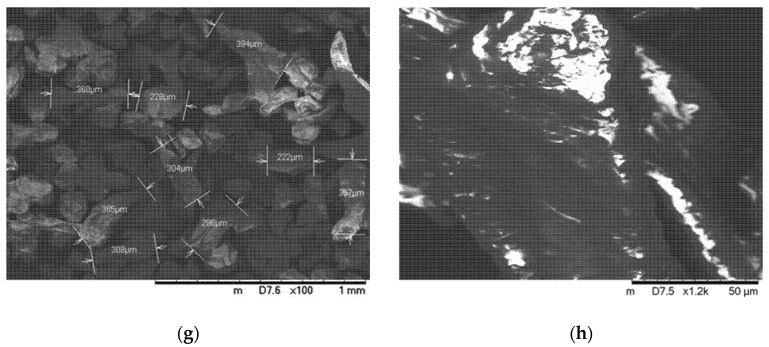
Determination of diameter of wet alginate microcapsules: (**a**) Light microscopic image of wet uncoated alginate microcapsules. The average size was 412.54 ± 28.83 µm. (**b**) SEM images of freeze-dried uncoated alginate microcapsules. (**c**,**d**) Shape and surface of uncoated alginate microcapsules, (**e**,**f**) chitosan-coated alginate microcapsules, and (**g**,**h**) Eudragit L100-55-coated microcapsules by SEM. The average size was determined by using 50 microcapsules.

The analysis of the shape and size of wet alginate microcapsules was performed by a light microscope after preparation. The mean value of wet alginate microcapsule diameter was 412.54 ± 28.83 µm when the nozzle size was 150 µm (see in Figure 3a). The shape of the wet microcapsules is isometric, nearly spherical, surrounded by a shell. After the freeze-drying of the microcapsules, the size of the solid particles was determined by SEM, as shown in Figure 3c,e,g. The average diameter of uncoated alginate microcapsules was 187.21 ± 24.36 µm, the chitosan-coated was 203.5 ± 31.98 µm, and the Eudragit-coated was 312.6 ± 50.36 µm (*n* = 50). The surface of the dried microcapsules is slightly rough, ribbed, which makes the surface irregular (Figure 3d). The shape of the dried microcapsules is almost isometric, in some places slightly elongated due to the formation of twins during drying (Figure 3c,e,g). The surface of the C-coated (chitosan-coated) microcapsules was less ribbed, while that of the E-coated (Eudragit L100-55 coated) samples was almost smooth.

### 3.3. Elemental Spectroscopy

**Figure 4:**
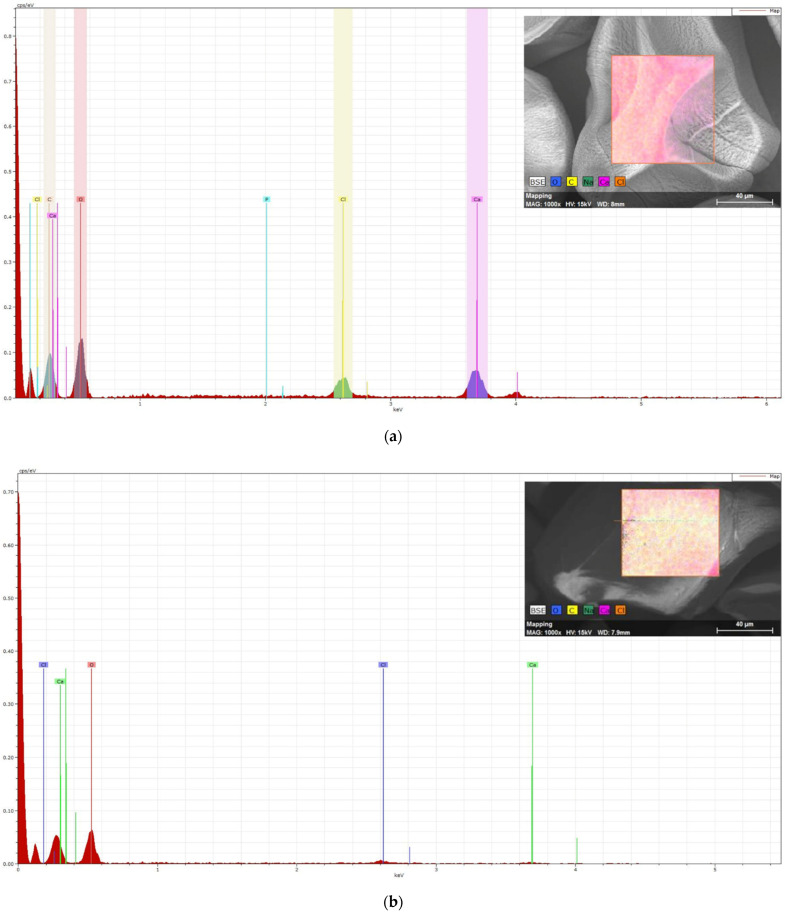

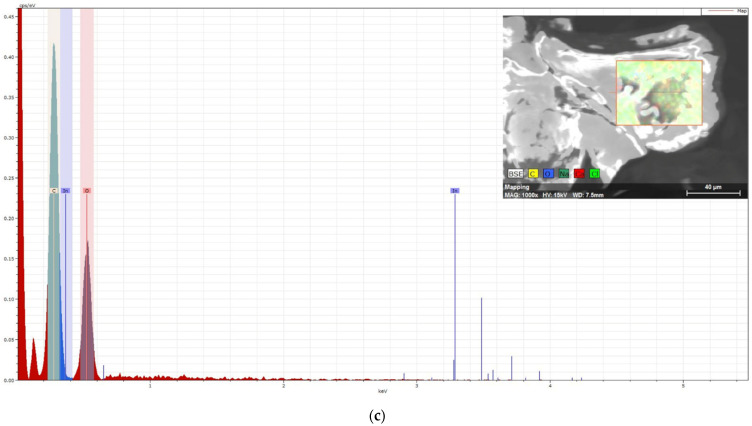
Elemental spectrum of uncoated alginate (**a**), chitosan-coated (**b**), and Eudragit L100-55-coated (**c**) microcapsule surface.

To check the effectiveness of the coating, the surface of the microcapsules was examined by photoelectron spectroscopy. Figure 4a shows a spectrum of the surface of the uncoated microcapsule, confirming the presence of calcium. Sodium alginate was used to prepare microcapsules; however, the spectrum did not show a sign of Na, indicating proper precipitation of alginate. Following chitosan coating, the signal intensity of calcium and chlorine was significantly reduced (see Figure 4b), suggesting effective chitosan coverage. The X-ray analysis confirmed that the main peaks in the chitosan spectrums are (C) and (O), which is the main elemental content of chitosan. In the spectrum of the surface of the microcapsule coated with Eudragit (on Figure 4c), the sign of calcium also disappeared, but the signs of C and O intensified. Based on the elemental analyzes performed from the surface, we can assume the appropriate and effective coating for both chitosan and Eudragit.

### 3.4. Dissolution Test of Microcapsule-Filled Gelatin Capsules

The effect of the pH of the solvent medium on the different coatings and the effect of the coating on the % survival of *Lactobacillus plantarum* were investigated. The dissolution was performed for 60 min in each dissolution medium. In consecutive dissolution in the pH 2.0 medium, the surface of the Eudragit L100-55-coated gelatin capsules was not swollen and no macroscopic sign of dissolution was seen. However, the uncoated gelatin capsules (GC) in acidic medium began to swell.

**Figure 5:**
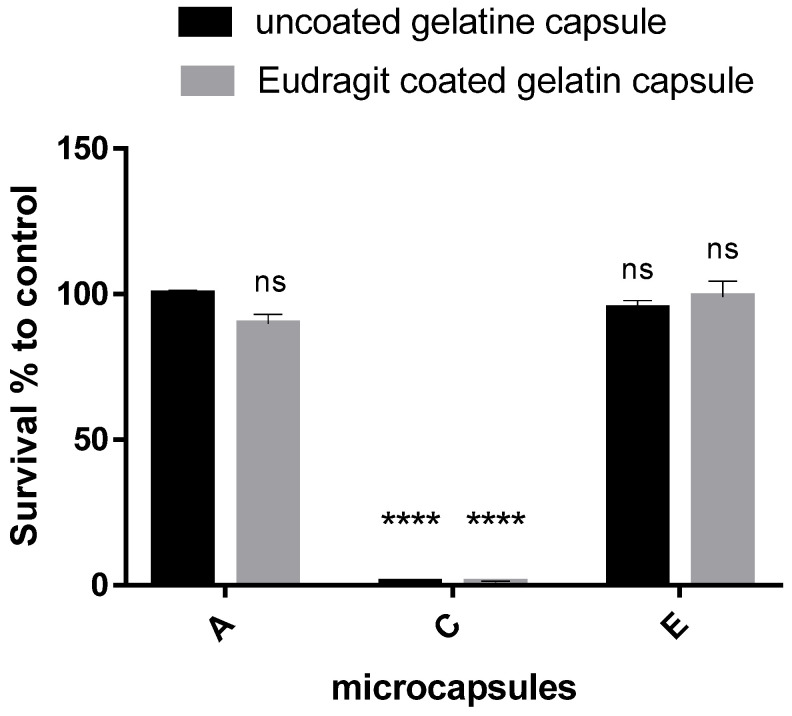
Survival compared to control (alginate uncoated microcapsules filled in uncoated gelatin capsule) of uncoated alginate (A), chitosan-coated (C), and Eudragit-coated (E) microcapsules filled in uncoated gelatin and Eudragit-coated gelatin capsule. Data are presented as means ± SDs, *n* = 3. Differences were considered significant at *p* < 0.05; **** *p* < 0.0001, and ns means not significant.

After 60 min, the pH 2.0 dissolution medium was changed to a pH 7.4 dissolution fluid, at which point the dissolution of the enteric-coated gelatin capsules also began and swelling of gelatin was observed. Figure 5 shows the pH 7.4 dissolution survival results compared to the control A filled into the uncoated GC. There was no significant difference in the survival of A filled into E-coated GC (89.81 ± 5.64) compared to the control. Similarly, no differences in survival were observed for E samples compared to controls (95.09 ± 4.73) or for gelatin capsule coating (E microcapsules in EGC 99.09 ± 9.27).

In the dissolution study of C, the viability of *Lactobacillus plantarum* was completely inhibited, so that the survival rate was practically zero when filled into both gelatin capsules.

### 3.5. Cell Release Profiles and Live/Dead Cell Determination from Coated and Uncoated Microcapsules (Number of Cells by Flow Cytometry)

**Figure 6:**
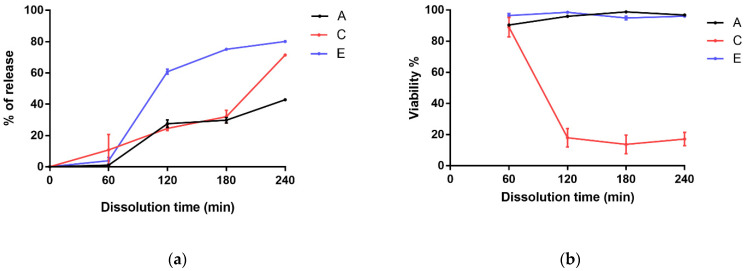
Release of *Lactobacillus plantarum* from microcapsules (**a**), and viability of released bacteria (**b**). Uncoated alginate (A), chitosan-coated (C), and Eudragit-coated (E) microcapsules were investigated in triplet. Data are presented as means ± SDs, *n* = 3.

In this experiment, the release of bacteria was studied depending on the pH and coating. Samples kept in pH 2.0 medium showed low cell counts after 60 min, with no significant difference (see Figure 6). After increasing the pH to 7.40, slow dissolution was measured in the uncoated microcapsules, and 42.86% of the initial bacterial concentration was released at 240 min. In the case of C-coated samples, similar dissolution kinetics were observed in the initial phase as for the alginate samples, but at 240 min, higher release (71.43%) was measured, as in the E-coated samples. For the E-coated samples, the fastest release was measured after adjusting the pH of the medium, resulting in the release of 80% of Lactobacilli. The initial cell concentration of the microencapsulation was 100%.

The release of bacterial cells after 60 min of pH change showed a significantly different mechanism. Dissolution of the chitosan coating in the pH 2.0 medium did not result in a significantly different dissolution profile compared to the uncoated alginate microcapsules, except for the last measurement point (240 min). From the surface of the E-coated microcapsules, the enterosolvent coat started to dissolve only after the pH change, which resulted in an explosive release of bacteria at 120 min, followed by a slower saturation phase. Bacterial cells were stained with SYTOX Green after sampling (60, 120, 180, and 240 min), and the live/dead cell ratio was examined. For the uncoated alginate and the E-coated microcapsules, no significant difference in viability was observed at sampling time points. However, samples coated with chitosan again showed a higher mortality rate after 60 min, which remained typical throughout the study. For the determination of viability, the sum of living and dead cells, i.e., the number of released bacteria, was considered to be 100% for each sample.

### 3.6. Determination of Dissolved Chitosan Coat

In view of the labeling efficiency and the labeled chitosan ratio in the coating solution, the concentration of chitosan in the dissolution medium was 0.043%, which was 431 ppm. The calculated value was determined after dissolution of microcapsules in pH 2.0 medium for 60 min. The chitosan coat was dissolved in the acidic media, then the pH of the dissolution medium was changed to 7.4 and samples were taken at 240 min. Deacetylation degree of chitosan was 75–85%, and the molecular weight was 50–190 kDa. The concentration of the dissolved chitosan coat from the surface of microcapsules was determined for the first time.

## 4. Discussion

The aim of our research and formulation development was to produce a probiotic containing a formulation used in animal feed with adequate stability. Using probiotic formulation as a premix component, it is usually mixed with a base with a high crude fiber content, which is extruded in high pressure steam and disaggregated to the appropriate size. Thus, in this technological process, the probiotic is exposed to a short-term heat load of around 80–100 °C. At the beginning of development, our aim was to embed the probiotic strain by microencapsulation and to apply a coating that is more resistant to heat.

The study began with the microencapsulation of three probiotic strains. All three probiotic microcapsules were also coated with chitosan, and the heat load of probiotic samples was investigated for the first time. The choice of the experimental conditions was selected based on the parameters previously used in the literature and the real extrusion parameters [25]. The same tendency was obtained for all three probiotic strains for uncoated and coated microcapsules because of each heat load. However, a distinction must be made between probiotic strains, since in the case of microcapsules containing *Enterococcus faecium*, the chitosan coating increased the thermotolerance compared to the uncoated microcapsules. For the other two strains (*Lactobacillus* and *Bifidobacterium*), the uncoated microcapsules showed better heat resistance, while those which were coated with chitosan were less viable. A similar result was published by Surono I. et al., and in their digestion test the survival percentage of *Enterococcus faecium* did not improve after microencapsulation, whereas *Lactobacillus plantarum* survival increased from 18.5% to 84.5% compared to free cells [6].

The aim of the following experiments was to investigate the results obtained with chitosan coating. *Lactobacillus plantarum* and *Bifidobacterium bifidum* showed a similar susceptibility pattern after the heat exposure; therefore, further experiments were performed focusing on *Lactobacillus plantarum.*

Chitosan coating-related viability was examined in the case of *Lactobacillus plantarum*-containing microcapsules. One group of microcapsules were coated with an intestinal soluble polymer, Eudragit L 100-55, to investigate the effect of different solubility coatings on the viability of *Lactobacillus plantarum*. The Eudragit L100-55 coating degradation is pH-dependent, and dissolved above pH 5.5 [28,29]. By this enterosolvent coating, the liberation of bacteria in acidic medium was avoided [30].

Commonly used artificial gastric juice is a pH 1–2 hydrochloric acid solution which sometimes contains sodium chloride as well. Diluted hydrochloric acid solution is more harmful for the bacteria than PBS. We wanted to exclude the viability changes of bacteria caused by the acidic medium; therefore, we chose more physiological PBS. The role of the acidic medium was only to dissolve the coating.

The quality and the efficiency of the coatings (C and E) were examined. SEM images present the surface differences and the surface irregularities of the uncoated, dried microcapsule. The surface became smoother with the chitosan coating; however, the smoothest surface was formed with the Eudargit L coating, where the cohesive coating was visible. The results of elemental analysis also confirmed that positively charged chitosan forms a complex with the negatively charged alginate on the surface of the C samples, which compensated the surface irregularities. In coating E, the methacrylate polymer completely covered the surface of the microcapsule, resulting in a 10% weight gain. To check the effectiveness of the coatings, three groups of microcapsules: uncoated alginate (A), chitosan-coated alginate (C), and Eudragit L100-coated (E) microcapsules, were filled into gelatin capsules. A half-part of the gelatin capsules was also coated with Eudragit L100-55, while the others were tested without the coating.

The dissolution of the uncoated and E-coated gelatin capsules in pH 7.4 again showed surprising results with chitosan-coated microcapsules. Regardless of the enteric coating of the gelatin capsule, viable bacteria could not be inoculated from the C-coated microcapsules. To reveal the result, another dissolution study was performed in which the release of *Lactobacillus plantarum* from the microcapsule, and the viability of the released bacteria, were examined by a flow cytometry.

Flow cytometry is a suitable method for the determination of the bacterial count [31,32]. In the cell release test, we investigated the released number of Lactobacilli. Chitosan dissolved in pH 2.0 medium, and afterwards the pH increased to 7.4, which caused the formation of a gel barrier on the surface of the microcapsules. Therefore, in chitosan-coated samples, the release of bacteria was hampered just in the last sampling time that increased the number of Lactobacilli, when it is probable that this gel barrier was disrupted. The Eudragit L100-55 (Evonic, Lot.No. B190304209) contains two emulsifiers: Sodium laurylsufate (0.7%) and Polysorbate 80 (2.3%). The concentration of emulsifiers did not decrease the viability of bacteria, but efficiently solubilized the alginate [33]. Thus, these excipients may accelerate the disintegration and increase the solubility of alginate, which resulted in the increased release of Lactobacilli.

SYTOX Green is a widely used fluorescent dye in the flow cytometric determination of live/dead cell ratios for the determination of bacterial viability [32,34]. A similar tendency was observed between the coating and *Lactobacillus* viability in the flow cytometric assay. The low proportion of viable microorganisms in the chitosan-coated samples confirmed the survival results of the CFU determination after the dissolution test.

All these results were compared with the published data, where no similar experience was described for the C-coated samples, and even the Lactobacilli had better viability in the C-coated samples. In addition to the results, it is worth comparing the method of the dissolution tests and the applied microcapsule/dissolution medium ratio. Thermotolerance was tested in the present study at a concentration of 15 mg microcapsule/mL of peptone water, which is a slightly more diluted concentration than reported in the literature. Cheow et al. applied 20 mg/mL of peptone water, but Jiang et al. used a more concentrated dissolution 100 mg/mL peptone water [13,25]. Dissolution and release tests performed in simulated gastric juice and intestinal fluid, and the applied microcapsule/medium ratio, were 10–20–100 mg/mL [35,36,37,38,39]. In this study, these dissolution and release experiments were performed with 6 mg of microcapsule and 50 mg/mL of the dissolution medium. The concentration of the microcapsule in the dissolution medium is an important factor due to the antibacterial effect of chitosan. Chitosan-coated probiotic microcapsules have been characterized in a number of studies, e.g., layer thickness, the formation of the alginate–chitosan–alginate structure, and the surface of chitosan-coated microcapsules. However, the concentration of chitosan in the dissolution medium has not yet been studied and compared with the MIC (minimum inhibitory concentration) for chitosan in the encapsulated probiotic strain. Some review publications deal with the evaluation of MIC values [40,41], and the MIC values reported for *Lactobacillus* strains are rather understudied. The only publication contains research on Lactobacillus strains [42]. The MIC values were reported for three strains for the different molecular weights of chitosan. The following MIC values were published for 224–28 kDa: *Lactobacillus plantarum* (0.05–0.05%), *Lactobacillus brevis* (>0.1–0.08%), and *Lactobacillus bulgaricus* (0.1–0.1%). The effect of the chitosan molecular weight and pH on MIC was investigated in the study. These MIC values were given for chitosan with a mass of 224–28 kDa, but the degree of acetylation of chitosan was not included, which also has an effect on the MIC value. In our experiments, the concentration of chitosan was determined according to the prescribed method (Method 2.9). We used equal microcapsule and dissolution medium ratio than we applied in flow cytometry experiments. Chitosan released from the microcapsule resulted in a concentration of 0.043% in the dissolution medium. Chitosan with a mass of 50–190 kDa was used for the formulation and in our experiments, which is of the same order of magnitude as given by No, H.K. et al. (228–28 kDa). Our results correlated with these published data in the research article and suggested that a strong inhibition was observed in chitosan-coated samples due to the dissolution near the MIC value.

Based on the compared data, it can be assumed that, in our experiments, the concentration of chitosan dissolved in the dissolution medium exceeds the MIC value of *Lactobacillus plantarum*. In addition to the amount of chitosan coating, several other factors affect the number of viable bacteria, such as the duration of the dissolution test and the duration of lyophilization. The duration of lyophilization, which significantly affects the initial bacteria count, has not been reported in most publications. In the thermotolerance test, the viability of *Enterococcus faecium* in the chitosan-coated samples was better than in uncoated alginate microcapsules. This may have resulted from the higher MIC value of chitosan. Therefore, the determination of the MIC values of the excipients in each study is necessary.

## 5. Conclusions

The application of probiotics in the form of microcapsules has several advantages in terms of bacterial viability, delivery, and release in appropriate amounts from the formulation. However, some factors need to be considered in the case of testing probiotic microcapsule formulations. The amount of coating agent released from each formulation, which was examined in our study, has not been tested yet. The application of a physiological microcapsule/gastrointestinal fluid ratio in the experimental system is important due to the antimicrobial activity of chitosan. MIC values have been published for various chitosan derivatives, confirming the use of chitosan in antibiotic preparations. However, the antimicrobial activity of chitosan has not been studied in terms of how it affects the viability of the released bacteria in the probiotic formulations in which it is a disadvantage. Further studies are needed to determine the viability-reducing effect of not only chitosan but also other similar coatings in a concentration-dependent manner. These studies may also influence the formulation development of the microcapsules (size, amount of coating) and the applied dose.

## Data Availability

The data presented in this study are available on request from the corresponding author.

## References

[1] Zamojska D., Nowak A., Nowak I., Macierzyńska-Piotrowska E. (2021). Probiotics and Postbiotics as Substitutes of Antibiotics in Farm Animals: A Review. Animals.

[2] Park H.J., Lee G.H., Jun J., Son M., Kang M.J. (2016). Multiple-unit tablet of probiotic bacteria for improved storage stability, acid tolerability, and in vivo intestinal protective effect. Drug Des. Devel. Ther..

[3] Stadler M., Viernstein H. (2003). Optimization of a formulation containing viable lactic acid bacteria. Int. J. Pharm..

[4] Arnal M.E., Denis S., Uriot O., Lambert C., Holowacz S., Paul F., Kuylle S., Pereira B., Alric M., Blanquet-Diot S. (2021). Impact of oral galenic formulations of Lactobacillus salivarius on probiotic survival and interactions with microbiota in human in vitro gut models. Benef. Microbes.

[5] Venema K., Verhoeven J., Beckman C., Keller D. (2020). Survival of a probiotic-containing product using capsule-within-capsule technology in an in vitro model of the stomach and small intestine (TIM-1). Benef. Microbes.

[6] Surono I., Verhoeven J., Verbruggen S., Venema K. (2018). Microencapsulation increases survival of the probiotic Lactobacillus plantarum IS-10506, but not Enterococcus faecium IS-27526 in a dynamic, computer-controlled in vitro model of the upper gastrointestinal tract. J. Appl. Microbiol..

[7] Ramos P.E., Cerqueira M.A., Teixeira J.A., Vicente A.A. (2018). Physiological protection of probiotic microcapsules by coatings. Crit. Rev. Food Sci. Nutr..

[8] Misra S., Pandey P., Dalbhagat C.G., Mishra H.N. (2022). Emerging Technologies and Coating Materials for Improved Probiotication in Food Products: A Review. Food Bioprocess Technol..

[9] Liao K., Cai J., Shi Z., Tian G., Yan D., Chen D. (2017). Effects of raw material extrusion and steam conditioning on feed pellet quality and nutrient digestibility of growing meat rabbits. Anim. Nutr. (Zhongguo Xu Mu Shou Yi Xue Hui).

[10] Itani K., Svihus B. (2019). Feed processing and structural components affect starch digestion dynamics in broiler chickens. Br. Poult. Sci..

[11] Rodrigues B.M., Olivo P.M., Osmari M.P., Vasconcellos R.S., Ribeiro L.B., Bankuti F.I., Pozza M.S.S. (2020). Microencapsulation of Probiotic Strains by Lyophilization Is Efficient in Maintaining the Viability of Microorganisms and Modulation of Fecal Microbiota in Cats. Int. J. Microbiol..

[12] Simon O., Jadamus A., Vahjen W. (2001). Probiotic feed additives-effectiveness and expected modes of action. J. Anim. Feed Sci..

[13] Deng Z., Hou K., Zhao J., Wang H. (2021). The Probiotic Properties of Lactic Acid Bacteria and Their Applications in Animal Husbandry. Curr. Microbiol..

[14] Rodklongtan A., La-ongkham O., Nitisinprasert S., Chitprasert P. (2014). Enhancement of Lactobacillus reuteri KUB-AC5 survival in broiler gastrointestinal tract by microencapsulation with alginate-chitosan semi-interpenetrating polymer networks. J. Appl. Microbiol..

[15] Ross G.R., Gusils C., Gonzalez S.N. (2008). Microencapsulation of probiotic strains for swine feeding. Biol. Pharm. Bull..

[16] Iyer C., Phillips M., Kailasapathy K. (2005). Release studies of Lactobacillus casei strain Shirota from chitosan-coated alginate-starch microcapsules in ex vivo porcine gastrointestinal contents. Lett. Appl. Microbiol..

[17] Zhang L., Li J., Yun T.T., Li A.K., Qi W.T., Liang X.X., Wang Y.W., Liu S. (2015). Evaluation of pilot-scale microencapsulation of probiotics and product effect on broilers1. J. Anim. Sci..

[18] Ding W.K., Shah N.P. (2007). Acid, bile, and heat tolerance of free and microencapsulated probiotic bacteria. J. Food Sci..

[19] Jiménez-Pranteda M.L., Poncelet D., Náder-Macías M.E., Arcos A., Aguilera M., Monteoliva-Sánchez M., Ramos-Cormenzana A. (2012). Stability of lactobacilli encapsulated in various microbial polymers. J. Biosci. Bioeng..

[20] Huq T., Fraschini C., Khan A., Riedl B., Bouchard J., Lacroix M. (2017). Alginate based nanocomposite for microencapsulation of probiotic: Effect of cellulose nanocrystal (CNC) and lecithin. Carbohydr. Polym..

[21] Huang X., Gänzle M., Zhang H., Zhao M., Fang Y., Nishinari K. (2021). Microencapsulation of probiotic lactobacilli with shellac as moisture barrier and to allow controlled release. J. Sci. Food Agric..

[22] Thangrongthong S., Puttarat N., Ladda B., Itthisoponkul T., Pinket W., Kasemwong K., Taweechotipatr M. (2020). Microencapsulation of probiotic Lactobacillus brevis ST-69 producing GABA using alginate supplemented with nanocrystalline starch. Food Sci. Biotechnol..

[23] Shaharuddin S., Muhamad I.I. (2015). Microencapsulation of alginate-immobilized bagasse with Lactobacillus rhamnosus NRRL 442: Enhancement of survivability and thermotolerance. Carbohydr. Polym..

[24] Cheow W.S., Kiew T.Y., Hadinoto K. (2014). Controlled release of Lactobacillus rhamnosus biofilm probiotics from alginate-locust bean gum microcapsules. Carbohydr. Polym..

[25] Cheow W.S., Hadinoto K. (2013). Biofilm-like Lactobacillus rhamnosus probiotics encapsulated in alginate and carrageenan microcapsules exhibiting enhanced thermotolerance and freeze-drying resistance. Biomacromolecules.

[26] Jiang T., Kim Y.-K., Singh B., Kang S.-K., Choi Y.-J., Cho C.-S. (2013). Effect of microencapsulation of Lactobacillus plantarum 25 into alginate/chitosan/alginate microcapsules on viability and cytokine induction. J. Nanosci. Nanotechnol..

[27] Cook M.T., Tzortzis G., Charalampopoulos D., Khutoryanskiy V.V. (2011). Production and evaluation of dry alginate-chitosan microcapsules as an enteric delivery vehicle for probiotic bacteria. Biomacromolecules.

[28] Tan E.W., Tan K.Y., Phang L.V., Kumar P.V., In L.L.A. (2019). Enhanced gastrointestinal survivability of recombinant Lactococcus lactis using a double coated mucoadhesive film approach. PLoS ONE.

[29] Zawari M., Poller B., Walker G., Pearson A., Hampton M., Carr A.C. (2019). Formulation of Broccoli Sprout Powder in Gastro-Resistant Capsules Protects against the Acidic pH of the Stomach In Vitro but Does Not Increase Isothiocyanate Bioavailability In Vivo. Antioxidants.

[30] Gartziandia O., Lasa A., Pedraz J.L., Miranda J., Portillo M.P., Igartua M., Hernández R.M. (2018). Preparation and Characterization of Resveratrol Loaded Pectin/Alginate Blend Gastro-Resistant Microparticles. Molecules.

[31] Bunthof C.J., Bloemen K., Breeuwer P., Rombouts F.M., Abee T. (2001). Flow cytometric assessment of viability of lactic acid bacteria. Appl. Environ. Microbiol..

[32] Kramer M., Obermajer N., Bogovic Matijasić B., Rogelj I., Kmetec V. (2009). Quantification of live and dead probiotic bacteria in lyophilised product by real-time PCR and by flow cytometry. Appl. Microbiol. Biotechnol..

[33] Matulyte I., Kasparaviciene G., Bernatoniene J. (2020). Development of New Formula Microcapsules from Nutmeg Essential Oil Using Sucrose Esters and Magnesium Aluminometasilicate. Pharmaceutics.

[34] Roth B.L., Poot M., Yue S.T., Millard P.J. (1997). Bacterial viability and antibiotic susceptibility testing with SYTOX green nucleic acid stain. Appl. Environ. Microbiol..

[35] Albadran H.A., Chatzifragkou A., Khutoryanskiy V.V., Charalampopoulos D. (2015). Stability of probiotic Lactobacillus plantarum in dry microcapsules under accelerated storage conditions. Food Res. Int..

[36] Mawad A., Helmy Y.A., Shalkami A.-G., Kathayat D., Rajashekara G. (2018). E. coli Nissle microencapsulation in alginate-chitosan nanoparticles and its effect on Campylobacter jejuni in vitro. Appl. Microbiol. Biotechnol..

[37] Chávarri M., Marañón I., Ares R., Ibáñez F.C., Marzo F., del Carmen Villarán M. (2010). Microencapsulation of a probiotic and prebiotic in alginate-chitosan capsules improves survival in simulated gastro-intestinal conditions. Int. J. Food Microbiol..

[38] Olivares A., Silva P., Altamirano C. (2017). Microencapsulation of probiotics by efficient vibration technology. J. Microencapsul..

[39] Ta L.P., Bujna E., Antal O., Ladányi M., Juhász R., Szécsi A., Kun S., Sudheer S., Gupta V.K., Nguyen Q.D. (2021). Effects of various polysaccharides (alginate, carrageenan, gums, chitosan) and their combination with prebiotic saccharides (resistant starch, lactosucrose, lactulose) on the encapsulation of probiotic bacteria Lactobacillus casei 01 strain. Int. J. Biol. Macromol..

[40] Sahariah P., Másson M. (2017). Antimicrobial Chitosan and Chitosan Derivatives: A Review of the Structure-Activity Relationship. Biomacromolecules.

[41] Verlee A., Mincke S., Stevens C.V. (2017). Recent developments in antibacterial and antifungal chitosan and its derivatives. Carbohydr. Polym..

[42] No H.K., Park N.Y., Lee S.H., Meyers S.P. (2002). Antibacterial activity of chitosans and chitosan oligomers with different molecular weights. Int. J. Food Microbiol..

